# Genome‐wide identification, gene expression and haplotype analysis of the *rhomboid‐like* gene family in wheat (*Triticum aestivum* L.)

**DOI:** 10.1002/tpg2.20435

**Published:** 2024-02-13

**Authors:** Yanyan Zhang, Xiaoya Huang, Long Zhang, Weidong Gao, Jingfu Ma, Tao Chen, Delong Yang

**Affiliations:** ^1^ State Key Laboratory of Aridland Crop Science Lanzhou China; ^2^ College of Life Science and Technology Gansu Agricultural University Lanzhou China; ^3^ College of Agronomy Gansu Agricultural University Lanzhou China

## Abstract

The *rhomboid‐like* (*RBL*) gene encodes serine protease, which plays an important role in the response to cell development and diverse stresses. However, genome‐wide identification, expression profiles, and haplotype analysis of the *RBL* family genes have not been performed in wheat (*Triticum aestivum* L.). This study investigated the phylogeny and diversity of the *RBL* family genes in the wheat genome through various approaches, including gene structure analysis, evolutionary relationship analysis, promoter *cis*‐acting element analysis, expression pattern analysis, and haplotype analysis. The 41 *TaRBL* genes were identified and divided into five subfamilies in the wheat genome. *RBL* family genes were expanded through segmented duplication and purification selection. The *cis*‐element analysis revealed their involvement in various stress responses and plant development. The results of RNA‐seq and quantitative real‐time‐PCR showed that *TaRBL* genes displayed higher expression levels in developing spike/grain and were differentially regulated under polyethylene glycol, NaCl, and abscisic acid treatments, indicating their roles in grain development and abiotic stress response. A kompetitive allele‐specific PCR molecular marker was developed to confirm the single nucleotide polymorphism of *TaRBL14a* gene in 263 wheat accessions. We found that the elite haplotype *TaRBL14a‐Hap2* showed a significantly higher 1000‐grain weight than *TaRBL14a‐Hap11* in at least three environments, and the *TaRBL14a‐Hap2* was positively selected in wheat breeding. The findings will provide a good insight into the evolutionary and functional characteristics of the *TaRBL* genes family in wheat and lay the foundation for future exploration of the regulatory mechanisms of *TaRBL* genes in plant growth and development, as well as their response to abiotic stresses.

## Core Ideas

- A total of 41 *TaRBL* genes were identified in the wheat genome.
- The wheat *RBL* family genes were expanded through segmented duplication and purification selection.
- *TaRBL* genes were generally highly expressed in panicles/grains.
- A kompetitive allele‐specific PCR molecular marker was developed for *TaRBL14a*.
- The haplotype *TaRBL14a‐Hap2* was favorable for improving grain weight.

**Abbreviations**
ABA: abscisic acid
DPA: days post anthesis
ER: endoplasmic reticulum
GL: grain length
GT: grain thickness
GW: grain width
*K*_a_
: non‐synonymous substitution rate
KASP: kompetitive allele‐specific PCR
*K*_s_
: synonymous substitution rate
MW: molecular weight
pI: isoelectric point
PEG: polyethylene glycol
SNP: single nucleotide polymorphism
TGW: 1000‐grain weight

## 1 INTRODUCTION

Regulated intramembrane proteolysis (RIP) regulates the transmembrane signaling transduction by splitting transmembrane proteins and releasing cytoplasmic fragments and is thus involved in various biological processes, such as cell differentiation, lipid metabolism, and stress response (Bölter et al., 2006; Brown et al., 2000). RIP proteases can be divided into three types, including aspartate proteases, S2P metalloproteases, and serine protease families (Brown et al., 2000; Rawson, 2002; Weihofen & Martoglio, 2003; Wolfe & Kopan, 2004). The *rhomboid‐like* (*RBL*) family genes encode a family of transmembrane serine proteases, which typically contain six or seven transmembrane domains and play vital roles in the intracellular signal transduction by modifying transmembrane protein substrates (Ha et al., 2013; Koonin et al., 2003; Lemberg & Freeman, 2007). RBL proteins have been reported to be involved in regulating various cytological processes, such as growth factor signaling transduction, endoplasmic reticulum (ER)‐associated protein degradation, and cell proliferation and death (Adrain & Cavadas, 2020; Adrain et al., 2011; Bock et al., 2022; Greenblatt et al., 2011; Kanaoka et al., 2022; Li et al., 2015; Urban et al., 2001).

The RBL protein was first discovered in Drosophila (*Drosophila melanogaster*) and named Rho‐1, which directly cleaved transforming growth factor α (TGFα) Spitz within its transmembrane domain and activated the epidermal growth factor receptor (EGFR) pathway in cell (Urban et al., 2001). Then, ubiquitin‐binding RBL4 was found to play a vital role in identifying and cleavage of unstable substrates in ER to promote their degradation (Fleig et al., 2012;. J. D. Knopf et al., 2020; Li et al., 2015).

In *Arabidopsis*, a total of seven *RBL* genes have been isolated and named *AtRBL1‐AtRBL7* (Kanaoka et al., 2005). AtRBL2 was found to cleave the *Drosophila* ligands Spitz and Keren, when expressed in mammalian cells, indicating that the proteolytic activity and substrate specificity of this family also conserved in plants (Kanaoka et al., 2005). Comparative proteomic analysis using double‐knockout plants lacking both chloroplast RBL proteins AtRBL8 and AtRBL9 showed a decreased amount of several chloroplast envelope proteins compared with the wild‐type plants (R. R. Knopf et al., 2012). The *RBL10* gene has been reported to be necessary for the processes of root growth, floral development, fertility, and photoprotection (Lavell et al., 2021; Thompson et al., 2012). A recent study showed that KOM, a Rhomboid serine protease protein, was involved in the regulation of meiocyte‐specific callose accumulation and pollen wall formation (Kanaoka et al., 2022). In addition, ER‐associated degradation factor Derlin‐1, as a rhomboid protease, was required for the dislocation of the mutant α‐1 antitrypsin from the ER (Greenblatt et al., 2011). The inhibition of the rice (*Oryza sativa* L.) *OsDER1* gene can activate the unfolded protein responses and the hypersensitivity to ER stress, resulting in powderiness and shrinkage of seeds (Qian et al., 2018). Several members of the *RBL* genes were also found to be involved in the abiotic stress responses. For example, the ER‐bound ANAC013 factor was cleaved by the RBL2 in the initial response to hypoxia in *Arabidopsis* (*Arabidopsis thaliana*) (Eysholdt‐Derzsó et al., 2023). A genome‐wide association analysis study reported that candidate genes for osmotic adjustment and drought traits included *RBL*, *DREB1*, and *SWEET* genes in durum wheat, suggesting that *RBL* genes could be involved in the regulation of the osmotic adjustment and drought stress in plants (Condorelli et al., 2022).

Wheat (*Triticum aestivum* L.) provides nearly 20% of the protein and calories in the human diet, so increasing wheat production is a priority for global food security (Calderini et al., 2021; Foulkes et al., 2011). *RBL* gene has been reported to be involved in plant growth and development, especially in influencing seed morphology in *Arabidopsis* and rice (Bölter et al., 2006; Qian et al., 2018; Thompson et al., 2012). However, the members and biological functions of the wheat *RBL* gene are poorly understood. In this study, the members of the *RBL* family genes were identified at the wheat genome‐wide level, and their physicochemical properties, gene structure, conserved domains, gene duplication, and expression patterns were analyzed. Among the wheat *RBL* gene family, a kompetitive allele‐specific PCR (KASP) molecular marker was developed for the *TaRBL14a* with a high expression level in spike/grain and the favorable haplotype, and temporal and spatial distribution was analyzed in the wheat breeding process. These results provide insights for further exploration of the function of *RBL* genes.

## 2 MATERIALS AND METHODS

### 2.1 Identification of *TaRBL* genes

The wheat genome data file was acquired from the Ensembl Plants database (http://plants.ensembl.org/info/website/ftp/index.html). The RBL conserved domain HMMER file (PF01694) as the query sequence downloaded from the Browse‐InterPro (http://ebi.ac.uk) database (Paysan‐Lafosse et al., 2023). The HMM search 3.0 software program was used to search for the whole genome protein sequence of wheat (threshold *E* < 1e‐5) to obtain the *TaRBL* candidate genes (Eddy, 2011). The reported members of the Rhomboid family in *Arabidopsis*, rice, and maize (*Zea mays* L.) were blasted as the reference species (Li et al., 2015). The redundant sequence in the HMM search and the BLAST results were removed. Subsequently, the NCBI‐CDD (https://www.ncbi.nlm.nih.gov/Structure/bwrpsb/bwrpsb.cgi) and SMART databases (https://smart.embl.de/) were used to confirm these protein sequences (Letunic et al., 2021; Marchler‐Bauer et al., 2005). Based on the *RBL* phylogenetic clustering in *Arabidopsis* and rice, the identified wheat *RBL* gene nomenclature was consistent with the description in previous reports (Li et al., 2015). Using the ExPASY server (https://web.expasy.org/compute_pi/) to calculate the molecular weight (MW) and theoretical isoelectric point (pI) of proteins in the wheat *TaRBL* genes family. The subcellular localization was predicted using the WoLFPSORT website (Table 1) (https://wolfpsort.hgc.jp/). The DeepTMHMM (https://dtu.biolib.com/DeepTMHMM), Phobius (https://phobius.sbc.su.se/), and CCTOP (https://cctop.ttk.hu/job/submit) databases were used to predict transmembrane helixes. There were false positive or ambiguous results in the algorithm applied to predict the transmembrane helix, so we adopted the “TM≥6” principle to provide a reasonable annotation for transmembrane prediction.

**Core Ideas**

- A total of 41 *TaRBL* genes were identified in the wheat genome.
- The wheat *RBL* family genes were expanded through segmented duplication and purification selection.
- *TaRBL* genes were generally highly expressed in panicles/grains.
- A kompetitive allele‐specific PCR molecular marker was developed for *TaRBL14a*.
- The haplotype *TaRBL14a‐Hap2* was favorable for improving grain weight.

**TABLE 1:** Characteristics of the *TaRBL* gene members in wheat

Name	Gene ID	Chromosome location	Size (aa)	pl	MW (kD)	Subcellular location
TaRBL3b1	TraesCS1A02G188900	1A:341872094…341873949	327	9.77	35.60	Plasma membrane
TaRBL3b2	TraesCS1B02G196600	1B:353945896…353947782	326	9.58	35.44	Plasma membrane
TaRBL3b3	TraesCS1D02G187500	1D:258978473…258980406	323	9.77	35.27	Plasma membrane
TaRBL3e2	TraesCS2A02G401000	2A:654874041…654876087	341	9.07	36.91	Plasma membrane
TaRBL3e3	TraesCS2B02G419100	2B:600707520…600709676	338	8.95	36.66	Plasma membrane
TaRBL3e4	TraesCS2D02G398500	2D:511527169…511529185	339	8.95	36.76	Plasma membrane
TaRBL4c	TraesCS3A02G071500	3A:44428992…44431556	374	8.39	40.76	Plasma membrane
TaRBL4b	TraesCS3B02G085300	3B:53812726…53815464	374	8.39	40.55	Plasma membrane
TaRBL4a	TraesCS3D02G070700	3D:31716241…31724528	305	8.82	33.02	Plasma membrane
TaRBL1a	TraesCS4A02G176100	4A:447320692…447324751	317	9.29	34.60	Plasma membrane
TaRBL3e1	TraesCS4A02G179600	4A:453299867…453307202	192	9.89	20.71	Vacuole
TaRBL1b	TraesCS4B02G141600	4B:187017000…187021129	385	9.70	41.92	Plasma membrane
TaRBL3d	TraesCS5D02G320400	5D:412599286…412602539	320	9.16	35.01	Plasma membrane
TaRBL3c1	TraesCS7A02G269900	7A:275960158…275970050	323	8.96	35.71	Plasma membrane
TaRBL3c2	TraesCS7B02G168100	7B:234905446…234911164	328	8.87	36.17	Plasma membrane
TaRBL3c3	TraesCS7D02G270400	7D:256185701…256191121	328	9.01	36.08	Plasma membrane
TaRBL10b	TraesCS2A02G566500	2A:765663084…765665593	314	9.53	33.70	Chloroplast
TaRBL10c	TraesCS2B02G630200	2B:801253452…801255821	292	9.77	31.60	Chloroplast
TaRBL10a	TraesCS2D02G576700	2D:640174317…640176964	347	9.80	37.49	Chloroplast
TaRBL10e	TraesCS5A02G236300	5A:451880134…451883804	344	10.81	36.92	Chloroplast
TaRBL10d	TraesCS5B02G234600	5B:412681434…412685170	344	10.69	36.97	Nucleus
TaRBL10f	TraesCS5D02G243300	5D:351468635…351476886	344	10.47	36.84	Nucleus
TaRBL11b	TraesCS1A02G127400	1A:154574636…154577511	278	9.61	30.54	Chloroplast
TaRBL11a	TraesCS1B02G147500	1B:212748274…212750997	209	9.12	23.16	Plasma membrane
TaRBL11c	TraesCS1D02G126800	1D:138345042…138347943	278	9.78	30.69	Chloroplast
TaRBL13a	TraesCS3B02G431900	3B:671015113…671015937	274	9.10	30.03	Vacuole
TaRBL13b	TraesCS3D02G393100	3B:508380832…508381647	271	9.10	29.75	Vacuole
TaRBL14e	TraesCS1A02G248200	1A:440183810…440187326	318	9.45	35.32	Chloroplast
TaRBL14d	TraesCS1B02G258700	1B:456184940…456188362	342	9.72	37.48	Chloroplast
TaRBL14c	TraesCS1D02G247600	1D:340557672…340562523	307	9.48	33.99	Chloroplast
TaRBL14a	TraesCS3A02G182700	3A:212087787…212090176	333	9.37	36.77	Plasma membrane
TaRBL14b	TraesCS3B02G212700	3B:252356857…252359300	333	9.08	36.45	Plasma membrane
TaRBL15b	TraesCS5A02G370500	5A:569863642…569869224	401	5.45	44.16	Endoplasmic reticulum
TaRBL15a	TraesCS5B02G372800	5B:550727559…550733169	386	5.84	42.48	Endoplasmic reticulum
TaRBL15c	TraesCS5D02G380000	5D:450657631…450663473	386	5.69	42.47	Endoplasmic reticulum
TaPARL1	TraesCS3A02G303000	3A:537485381…537498089	325	11.46	35.39	Chloroplast
TaPARL2	TraesCS3B02G333300	3B:539660545…539672124	327	11.59	35.53	Chloroplast
TaPARL3	TraesCS3D02G298800	3D:412828008…412834724	325	11.62	35.27	Chloroplast
TaPARL4	TraesCS6A02G369500	6A:594979250…594982971	307	11.25	32.36	Mitochondrion
TaPARL5	TraesCS6B02G405700	6B:680726871…680729775	226	9.85	24.74	Vacuole
TaPARL6	TraesCS6D02G352900	6D:448902784…448906771	349	10.92	37.22	Chloroplast

Abbreviation: MW, molecular weight.

### 2.2 Sequence alignment and phylogenetic analysis of *TaRBL* genes

The MUSCLE method in soft MEGA 11.0 was applied to multiplex the sequence alignment of RBL protein sequences in *Arabidopsis*, rice, maize, and wheat. The neighbor joining method was used to construct a phylogenetic tree with 1000 bootstrap replications (Tamura et al., 2013). Finally, the tree was visualized with the online tool iTOL (https://itol.embl.de/upload.cgi). According to this tree, *RBL* families can be divided into five subgroups and named as RhoA, RhoB, RhoC, RhoD, and RAPL (Li et al., 2015).

### 2.3 Gene structure, motif, and domain analysis of *TaRBL* members

The GSDS server (http://gsds.gao‐lab.org/) was used to compare the genomes of *TaRBL* genes and their corresponding CDS sequences and to analyze the structural schematic diagram of the *TaRBL* genes. Using the conserved motif to identify the TaRBL protein via the MEME v5.5.3 (MEME‐Submission form (meme‐suite.org)) with the motif number set to 20 (Table S1) (Bailey et al., 2009). The function domain of TaRBL proteins was identified using the SMART website (SMART: Main Page (embl.de)). The visual plotting was constructed by the TBtools software (Chen et al., 2020).

### 2.4 Chromosome location, gene duplication, and collinearity analysis of *TaRBL* genes

The physical location of the *TaRBL* genes was obtained from the wheat genome annotation information (gff3), and the *TaRBL* genes were mapped on the wheat chromosome. The segmental duplication and tandem repeat events for *TaRBL* genes family were analyzed through MCScanX program in the TBtools software. The Multiple Synteny Plot module of TBtools was employed to conduct the collinearity analysis of *RBL* genes in wheat, *Arabidopsis*, rice, and maize. The synonymous substitution rates (*K*_s_) and the non‐synonymous substitution rates (*K*_a_) of duplicate gene pairs were calculated using the TBtools software (Table S2). Here, the *K*_a_/*K*_s_ ratio = 1 means neutral mutation, *K*_a_/*K*_s_ < 1 represents purifying selection, and *K*_a_/*K*_s_ > 1 represents the trend evolution accelerator with positive selection (Zhang et al., 2006).

### 2.5 Analysis of the *cis*‐regulatory elements in the promoter of *TaRBL* genes

The DNA sequence of *TaRBL* genes initiation codon (ATG) upstream 2000 bp was extracted from the whole wheat genome sequence through the TBtools software. They were submitted to the online database PlantCARE (http://bioinformatics.psb.ugent.be/webtools/plantcare/html/) for analyzing *cis*‐acting elements.

### 2.6 Expression profiles analysis of *TaRBL* genes

The RNA‐Seq data of *TaRBL* genes in different tissues/organs of the wheat variety of Chinese spring at different developmental periods were downloaded from the WheatOmics1.0 (http://202.194.139.32/expression/wheat.html). The differential expression of *TaRBL* family genes was analyzed, and then the data were visualized and mapped based on the TBtools software.

### 2.7 Plant growth and abiotic stress treatments

The seeds of the winter wheat variety Jinmai 47 were cultured in Hoagland nutrient solution after disinfection with sodium hypochlorite and rinsing with distilled water, with a photoperiod of 16 h light/8 h darkness and a temperature of 25°C. The Hoagland nutrient solution was changed every 3 days to ensure a consistent supply of nutrients. When the seedlings had grown to 12 days, they were treated with 20% polyethylene glycol (PEG) 6000, 200 mM NaCl, and 100 μM abscisic acid (ABA). The first leaves of wheat seedlings were taken at 0, 6, 12, 24, and 48 h after stressed treatments, placed in the liquid nitrogen for quick freezing, and then stored in a −80°C freezer. Each treatment was replicated three times.

The wheat seedlings, roots, stems, leaves, and young spikes at the booting stage, grains at 5, 10, 15, 20, 25, and 30 days post anthesis (DPA), were collected for RNA extraction and tissue expression analysis.

The 263 wheat accessions (Table S3) used for the KASP marker development and association analysis were grown in Tongwei farm station (35°11′N, 105°19′E, altitude 1750 m) in 2021, Tongwei and Zhuanglang farm station (35°21′N, 105°58′E, altitude 2110 m) in 2022, and Tianshui farm station (34°34′N, 105°53′E, altitude 1550 m) and Zhuanglang in 2023, respectively. All wheat accessions were sown in late September and harvested in early July of the following year. A randomized complete block design was conducted with three replications, where the row length of each plot was 1 m and the row spacing was 20 cm, and 30 seeds were sown in each row. Local wheat cultivation practices were considered in field management. After harvesting, two hundred seeds for each cultivar were used to measure grain length (GL), grain width (GW), and 1000‐grain weight (TGW) by an image analysis system using the SC‐G wheat grain appearance quality (Hangzhou WSeen Detection Technology Co., Ltd.). All measurements were conducted with three biological replicates.

### 2.8 Quantitative real‐time‐PCR (qRT‐PCR) analysis of *TaRBL* genes

The TIANGEN Plant Tissue RNA Rapid Extraction Kit was used to extract total RNA from collected samples and determine RNA concentration by ultra microphotometer. The synthesis of the first strand of cDNA using FastKing gDNA Dispelling RT SuperMix. FastReal qPCR PreMix (SYBR Green) was used to detect the changes of the relative expression of six genes in different tissues and under different stress treatments by qRT‐PCR analysis. Wheat *TaGADPH* was used as a reference gene for wheat tissue development expression analysis, and wheat *TaActin* was used as reference gene for wheat stress expression analysis (L. Guo et al., 2022; Mei et al., 2022). The primers for qRT‐PCR were listed in Table S4. The relative expression levels of six *TaRBL* genes were calculated using the 2^−ΔΔCT^ method (Schmittgen & Livak, 2008). All quantifications were performed on three biological replicates.

### 2.9 Polymorphism assays of *TaRBL14a*

Based on the wheat resequencing data in the WheatUnion1.0 database (http://wheat.cau.edu.cn/WheatUnion/b_4/), the mutation sites of *TaRBL14a* in different wheat cultivars were obtained (Cheng et al., 2019; Dong et al., 2022; W. Guo et al., 2020; Hao et al., 2020; Liu et al., 2021; Walkowiak et al., 2020; Wang et al., 2020; Yang et al., 2022; Zhou et al., 2020). The PlantPAN3.0 (http://plantpan.itps.ncku.edu.tw) was used to predict the *cis*‐acting element in the 2000 bp promoter region of *TaRBL14a* (Chow et al., 2019). To further confirm this result, the DNA of 10 wheat accessions containing two haplotypes was extracted using the cetyltrimethylammonium bromide method (Stewart & Via, 1993). The specific primers were designed to amplify the sequence of the single nucleotide polymorphism (SNP) site located in the promoter region (Table S4). The PCR was performed in a total volume of 15 μL consisting of 7.5 μL 2*Red Taq MasterMix, 1 μL each of forward and reverse primer (10 μM), 1.5 μL DNA (200 ng μL^−1^), and 4 μL ddH2O. The PCR conditions were as follows: 94°C for 5 min, 35 cycles of 94°C for 30 s, 58°C for 30 s, 72°C for 1 min, and a final extension of 72°C for 5 min. The PCR products were checked on agarose gel (1%), and the desired bands were purified using the TIANgel Purification Kit (TIANGEN) before gene sequencing (Sangon Biotech).

### 2.10 KASP marker development and association analysis

The SNP (C/G) at position −779 bp in the promoter of *TaRBL14a* was converted into KASP marker for genotyping of 260 wheat accessions from different provinces in China and three American wheat accessions (Table S3). Two reverse primers and one forward primer were designed for the TaRBL14a‐KASP marker using the WheatOmics database (Table S4). The KASP assays were tested in a 96‐well plate with 4 μL reaction volume containing 2 μL of 2× KASP master mix, 1 μL of SNP primer mix (4×), and 1 μL of DNA template. The PCR conditions were as follows: hot start at 94°C for 15 min, followed by 10 touchdown cycles (94°C for 20 s; touchdown at 61°C initially and decreasing by −0.6°C per cycle for 40 s), followed by 35 cycles of annealing (94°C for 20 s and 55°C for 45 s). The fluorescence signal was detected and read using Kluster Caller software (v. 3.4.1.36; https://www.biosearchtech.com).

The grain‐related phenotypic data of 111 wheat accessions from the published study (Ma et al., 2016), 260 wheat accessions from different provinces in China, and three American accessions in this study were used for association analysis using TASSEL5.1 software (Tables S5 and S6). A one‐way analysis of variance was performed to analyze significant differences using SPSS 22.0 (IBM Corporation). The geographical distribution of the two haplotypes of *TaRBL14a* in China was analyzed with 301 wheat accessions from the WheatUnion database and 224 wheat accessions collected from different provinces in China (Table S7). The selection of the two haplotypes of *TaRBL14a* in breeding history in China was analyzed using 117 wheat accessions from the WheatUnion database and 204 wheat accessions collected from different provinces in China (Table S8).

## 3 RESULTS

### 3.1 Identification of *RBL* members in wheat

In this study, a total of 41 wheat *TaRBL* genes were identified from the wheat genome. They were renamed as *TaRBL1a* to *TaPARL6* in order according to their position on the chromosome and the *Arabidopsis* nomenclature. The analysis of the physicochemical properties of TaRBL proteins showed that the protein length ranged from 192 to 401 aa, the protein MW ranged from 20.71 to 44.16 KDa, and the pI varied from 5.45 to 11.62. In addition, the subcellular prediction showed that TaRBL proteins were distributed in the plasma membrane, chloroplasts, ER, or vacuoles, indicating that *TaRBL* genes could play different roles in different biological processes (Table 1).

### 3.2 Phylogenetic analysis of *TaRBL* genes

**FIGURE 1:**
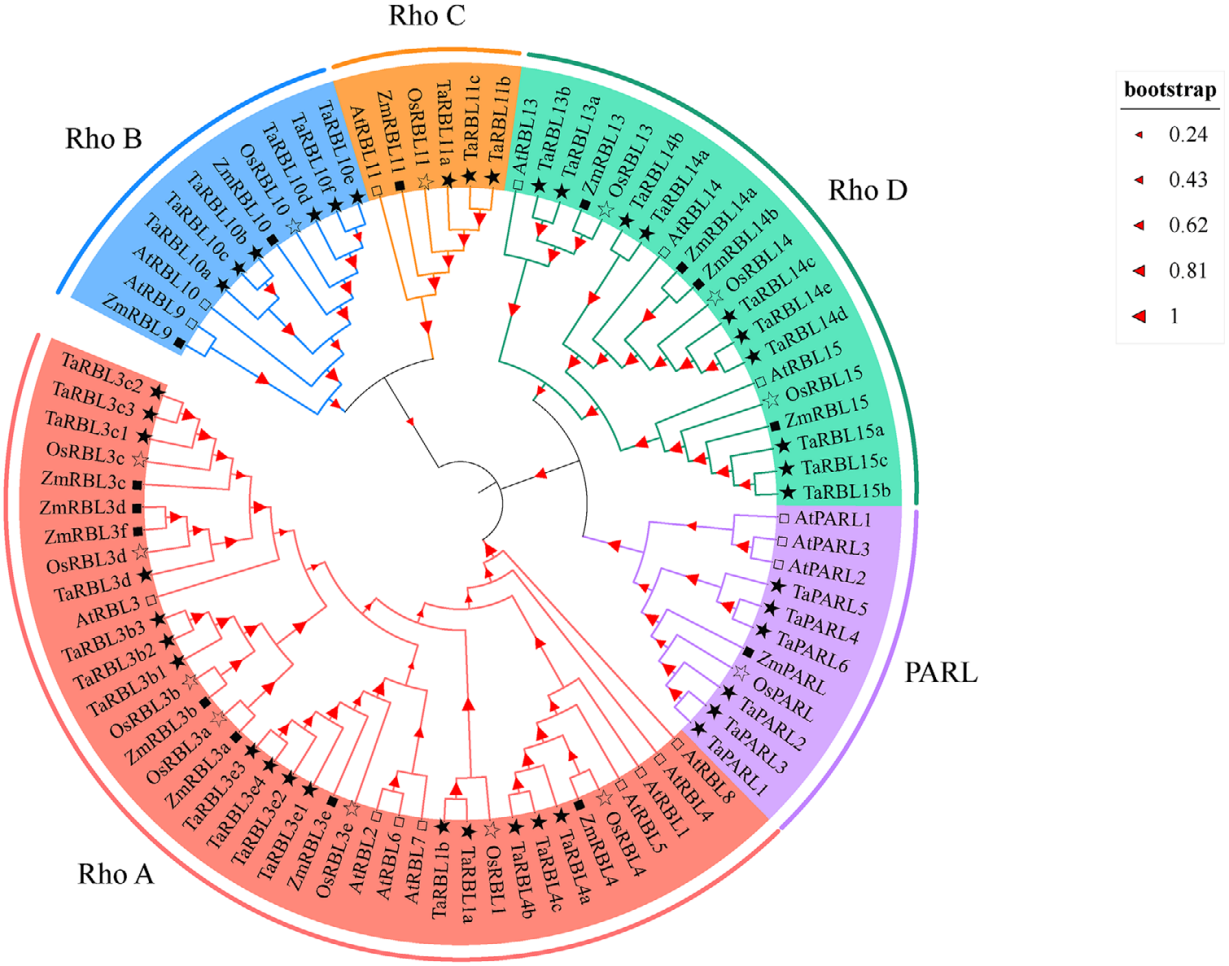
Phylogenetic tree of *rhomboid‐like* (RBLs) proteins from *Triticum aestivum* (41), *Oryza sativa* (13), *Zea mays* (15), and *Arabidopsis thaliana* (17). RhoA, RhoB, RhoC, RhoD, and RAPL represent different subfamilies, respectively.

The construction of the phylogenetic tree can further study the conservation of genes in the process of evolution. To analyze the evolutionary relationship of the *RBL* genes in wheat, we conducted a Neighbor‐Joining analysis with the RBL protein sequences encoded by *RBL* genes. The results showed that RBL proteins were divided into five subfamilies: RhoA, RhoB, RhoC, RhoD, and RAPL (Figure 1). In addition, the gene duplication and missing were identified mainly in the RhoA subfamily.

### 3.3 Gene structure and conserved motif analysis of *TaRBL* family members

**FIGURE 2:**
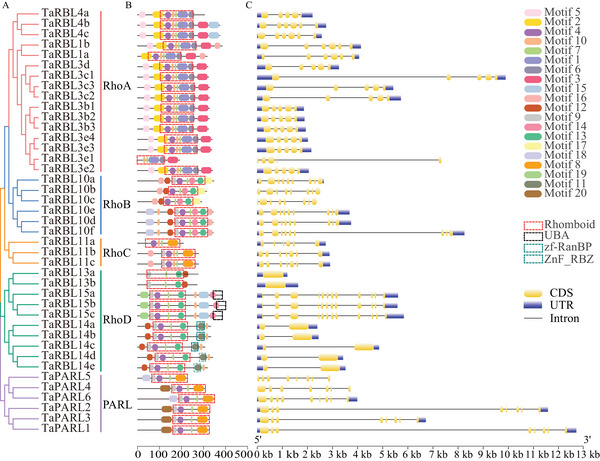
Phylogenetic relationships, conserved protein motifs and gene structures of the *TaRBLs*. (A) The phylogenetic tree was constructed based on the full‐length sequences of wheat rhomboid‐like (RBL) proteins. (B) The conserved motifs and functional domain of TaRBL proteins. (C) The Exon‐intron structures of *TaRBL* genes. UTR, untranslated region.

The exon‐intron structure of *TaRBL* family members was analyzed using GSDS to gain further insights into the *RBL* gene family. The results revealed that the *TaRBL* genes consist of 2–13 exons, with most members within the same subfamily exhibiting similarities in terms of exon length and intron number (Figure 2). Furthermore, the MEME software was applied to detect the motif composition, and the SMART database was employed to analyze the functional domain of RBL proteins in wheat. At least 20 motifs and four domains were identified successfully (Figure 2; Table S1). Among them, the Rhomboid domain is the main domain of RBL protein, which was identified in all TaRBL proteins. TaRBL proteins in the same subfamily had similar motifs and distributions, indicating functional similarity among members of the same subfamily. During evolution, different subfamilies could undergo divergence. For instance, TaRBL14s had an RBZ/RanBP‐type zinc finger domain at its carboxyl terminus, and TaRBL15s had a ubiquitin‐binding correlation motif (UBA). Therefore, the zinc finger structure of TaRBL14s could enable it to interact with other proteins or DNA, while TaRBL15s likely played a role in ubiquitinated degradation. All RBL family members comprised six or seven conserved transmembrane helices (TMHS). Of these, the histidine residues and GxxxG motifs in TMH6, which played an important role in protein structure and function (Figure S1). The serine in TMH4 and histidine in TMH6 were crucial for the enzymatically active sites required for proteolytic activity and were conserved in most RBL proteins, indicating their potential proteolytic activity (Figure S1).

### 3.4 Chromosome localization, gene duplication, and collinearity analysis of *TaRBL* family genes

**FIGURE 3:**
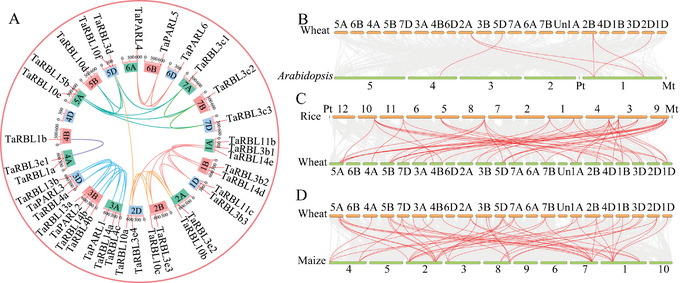
Collinearity analysis of *RBL* genes. (A) Duplicated gene pairs in the wheat genome. (B) Colinearity analysis of *TaRBL* genes with *Arabidopsis* genome. (C) Colinearity analysis of *TaRBL* genes with rice genome. (D) Colinearity analysis of *TaRBL* genes with maize genome. Duplicated gene pairs are linked by lines with the corresponding color. Gray lines indicate collinear blocks within the wheat genome and other plant genomes, and the red curve indicates *RBL* genes with collinearity.

The 41 *TaRBL* genes were unevenly localized in 20 chromosomes in wheat, except for 4D (Figure S2). The gene duplication was found in the genome expansion and function acquisition during the wheat polyploidization. Here, 32 segmental duplication gene pairs were identified in the wheat *TaRBL* family genes. Most of them belonged to the RhoA subfamily without the tandem duplication (Figure 3A). To explore the collinearity between the *TaRBL* genes of wheat and other species, we used the MCScanX software to analyze the collinear relationship of *RBL* genes between wheat and *Arabidopsis* (Figure 3B), rice (Figure 3C), and maize (Figure 3D). The results showed that wheat *TaRBL* had six homology genes with *Arabidopsis*, 49 homology genes with rice, and 52 homology genes with maize. These results were highly consistent with the phylogenetic analysis using the protein sequences from wheat, rice, and *Arabidopsis*, with collinear gene pairs belonging to the same subfamily. This indicated that most *TaRBL* genes were conservative among plants. The *K*_a_/*K*_s_ ratio of duplicated genes was calculated as a marker of selection pressure. The *K*_a_/*K*_s_ results showed that duplicated gene pairs in the *TaRBL* family were all less than 1, except for one pair (*TaPARL1* and *TaPARL3*), indicating that the *TaRBL* genes were mainly driven by purification selection pressure after duplicated events (Table S2).

### 3.5 *Cis*‐acting regulatory elements in the promoters of *TaRBL* genes

The 2000 bp upstream sequences of 41 *TaRBL* genes were extracted by the TBtools software and then submitted to the PlantCARE platform for the *cis*‐acting element prediction. As a result, 104 *cis*‐acting elements were identified in the promoter regions on *TaRBL* genes, including light‐responsive, development‐related, phytohormone‐responsive, stress‐responsive elements, and other elements with unknown function (Figure S3). Among them, MYC/MYB, CAT‐box, CCGTCC‐box, and ABRE elements related to the responses to plant development and abiotic stresses were found in almost all *TaRBL* genes (Figures S4–S6). These elements had been widely reported to be involved in the control of circadian rhythms, flowering time, hypocotyl elongation, and stress tolerance, suggesting that *TaRBL* genes could be involved in plant growth, development, and abiotic stress responses.

### 3.6 Analysis of the expression patterns of *TaRBL* genes in different organs

To preliminarily investigate the function of *TaRBL* genes, the transcriptomic data acquired from the WheatOmics 1.0 database were used to analyze the expression level of *TaRBL* genes in various organs (root, stem, leaf, spike, and grain). The results showed that 41 *TaRBL* genes exhibited different expression patterns in different organs (Figure S7). Of these, 25 *TaRBL* genes were highly expressed in the spike or grain, while eight *TaRBL* genes were highly expressed in the leaves. The *TaRBL* genes of the same subfamily generally showed similar expression patterns. For instance, both RhoD and PARL subfamily genes were highly expressed in spikes, while RhoA subfamily genes displayed a wide expression pattern in roots, stems, leaves, and spike/grain, except for *TaRBL4* specifically expressed in spike. These results indicated that *TaRBL* genes in the same subfamily could be functionally conserved.

**FIGURE 4:**
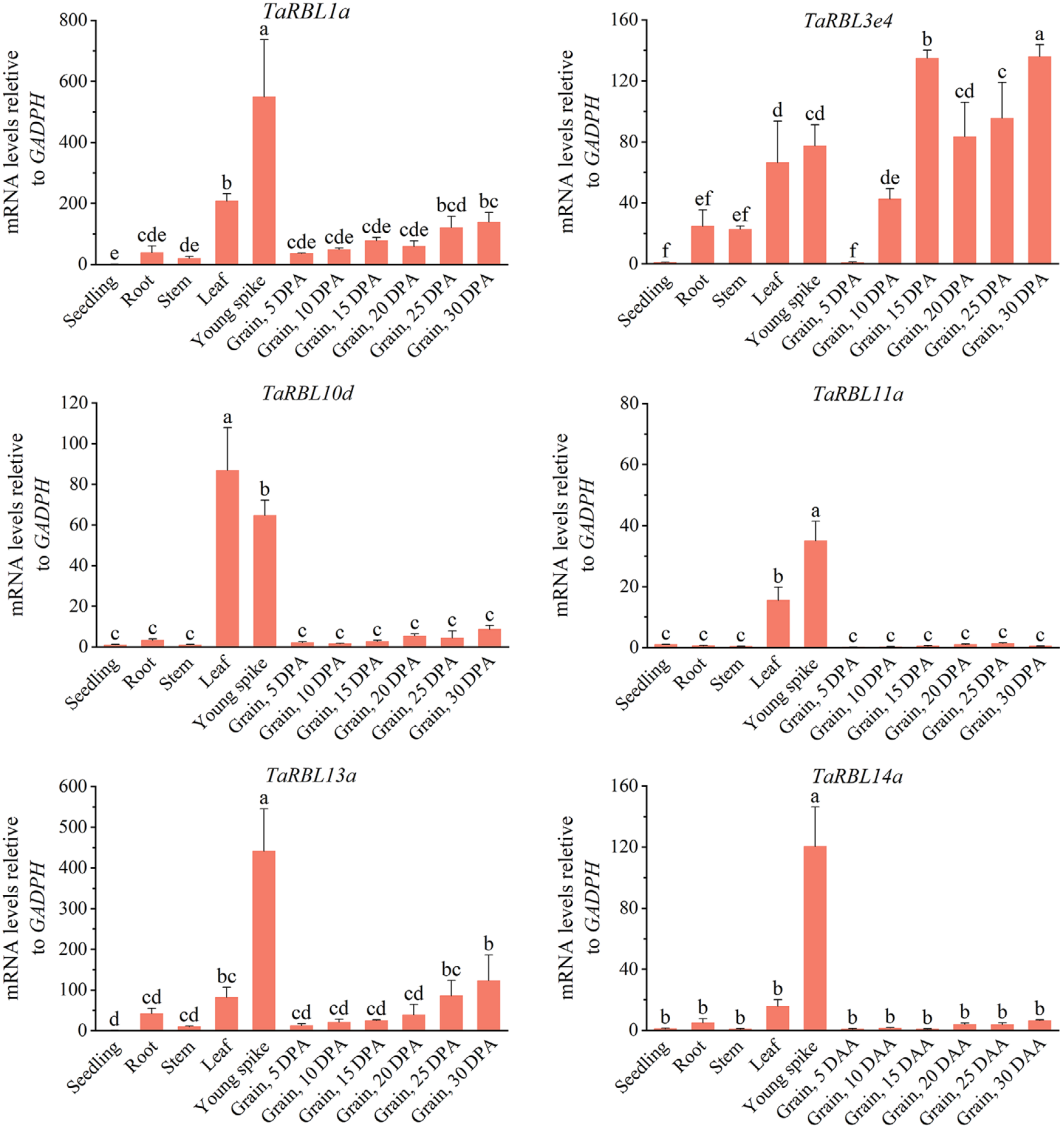
The quantitative real‐time‐PCR (qRT‐PCR) analysis of expression patterns of *TaRBLs* at different organs. The RNA extracted from seedlings, roots, stems, leaves, and young spike at the booting stage, grains at 5, 10, 15, 20, 25, and 30 days post anthesis (DPA) were used for inverse transcription and further analysis. The relative expression levels in control sample (CK Seedling) were normalized to 1. Data are the mean ± standard errors of three independent replicates.

To verify the results acquired from RNA‐Seq data, we selected six *TaRBL* genes from different subfamilies for qRT‐PCR analysis (Table S4). The expression levels of these genes were measured in seedlings, roots, stems, leaves, young spikes, and grains at 5, 10, 15, 20, 25, and 30 DPA by the qRT‐PCR determination (Figure 4). The qRT‐PCR results showed that all these genes were generally highly expressed in young spike, and their expression levels in grain at different stages were gradually increased after anthesis. These results indicated that *TaRBL* genes could participate in the regulation of spike and grain development.

### 3.7 Analysis of the expression level of *TaRBL* genes under different abiotic‐stressed treatments

**FIGURE 5:**
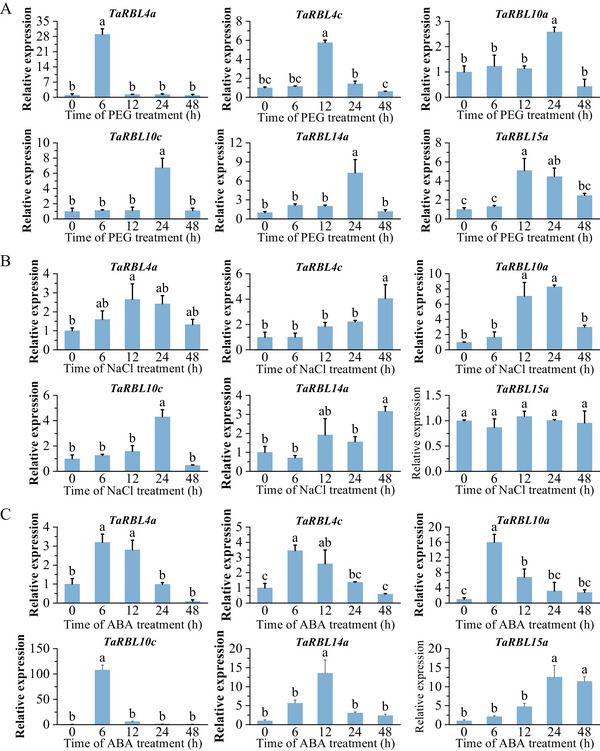
qRT‐PCR analysis of selected *TaRBL* genes under PEG6000, NaCl and ABA treatment. (A) Relative expression patterns of *TaRBL* genes in leaves after PEG 6000 treatment. (B) Relative expression patterns of *TaRBL* genes in leaves after NaCl treatment. (C) Relative expression patterns of *TaRBL* genes in leaves after ABA treatment. The relative expression levels in control sample (CK 0 h) were normalized to 1. Data are the mean ± standard errors of three independent replicates. ABA, abscisic acid; PEG, polyethylene glycol.

The results of the *cis*‐acting elements analysis showed that *TaRBL* genes were involved in the response to abiotic stresses. To understand the response of *TaRBL* genes under abiotic stress, qRT‐PCR was used to investigate the expression level of six genes treated with 20% PEG 6000, 200 mM NaCl, and 100 μM ABA (Figure 5). The transcript of most of these genes was induced under 20% PEG 6000 treatment. The expression levels of *TaRBL4a*, *TaRBL10a*, and *TaRBL10c* gradually increased after reaching a peak at 12 or 24 h and then decreased. The expression level of *TaRBL4c* was continuously increased from 0 to 48 h, while *TaRBL15a* did not show significant up‐regulation after 20% PEG 6000 treatment. Under 200 mM NaCl treatment, all *TaRBL* genes showed a significant up‐regulation expression pattern. The expression level of these genes was gradually increased and reached the peaks at 6, 12, or 24 h. As ABA response‐related *cis*‐elements were also identified in most *TaRBL* genes (Figure S6), the expression levels of these genes under 100 μM ABA treatment were further analyzed via qRT‐PCR. After 6 h of ABA treatment, the expression levels of all six *TaRBL* genes showed an up‐regulation trend. Notably, *TaRBL10c* displayed a peak expression level approximately 100‐fold higher than that of the control before rapidly declining. This suggested that *TaRBL* family genes could play an important role in abiotic stress response.

### 3.8 Association analysis of *TaRBL14a* and grain‐related traits in wheat

**FIGURE 6:**
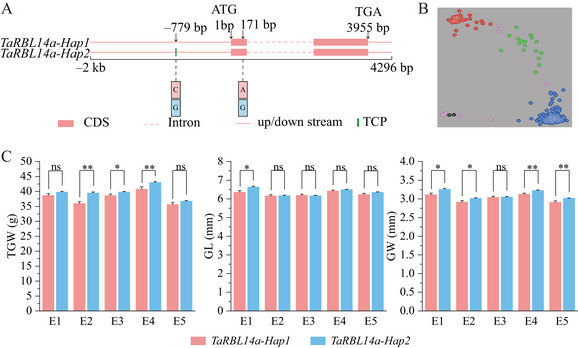
Effects of *TaRBL14a* haplotypes on grain phenotypic traits. (A) The distribution of main *cis*‐regulatory elements contained SNPs site in the promoter region of the two haplotypes of *TaRBL14a* gene. (B) A TaRBL14a‐KSAP marker developed based on −779 bp (C/G). The blue circles represent allele (G), the red circles represent allele (C), the green circles represent the heterozygous C/G allele, the pink and black circles represent missing values. (C) Association of *TaRBL14a‐Hap1* and *TaRBL14a‐Hap2* with 1000‐grain weight (TGW), grain length (GL), and grain width (GW) of 263 wheat accessions at five environments allelic. E1–E5 indicates Tongwei farm station, Gansu, China (35°11′N, 105°19′E, altitude 1750 m) in 2021, Tongwei farm station and Zhuanglang farm station (35°21′N, 105°58′E, altitude 2110 m) in 2022, and Tianshui farm station (34°34′N, 105°53′E, altitude 1550 m) and Zhuanglang in 2023. SNP, single nucleotide polymorphism; TGA, termination codon. *p < 0.05; **p < 0.01.

Based on the qRT‐PCR results, *TaRBL14a* exhibited specific expression in the young spike, indicating its potential role in the development of spike and grain. To uncover the naturally allelic variations of *TaRBL14a* in wheat, we detected the polymorphisms of the *TaRBL14a* promoter 2000 bp DNA fragment and coding region by using 619 varieties from the WheatUnion website. We detected one SNP in each of the promoter region and the coding region of *TaRBL14a* (Figure 6A). Based on two SNPs, two haplotypes of *TaRBL14a* were identified in these wheat collections, namely *TaRBL14a‐Hap1* and *TaRBL14a‐Hap2*. A specific primer was designed to amplify the SNP sequences of 10 wheat germplasm promoter regions containing two haplotypes. The SNP sequences of these materials were consistent with the resequencing results of the WheatUnion database (Figure S8). To detect the effect of *TaRBL14a* allele variation on yield‐related traits in wheat, the phenotypic average of grain traits between *TaRBL14a* allele variations was compared using 111 varieties (Table S5 and S6). *TaRBL14a‐Hap2* had significantly higher TGW, GL, GW, and grain thickness than *TaRBL14a‐Hap1* (Figure S9). A KASP marker was developed at −779 bp (C/G) in the *TaRBL14a* promoter region to distinguish these two haplotypes (Figure 6B). This KASP marker was further validated in 263 wheat accessions. Significant association (*p* < 0.05) between two *TaRBL14a* haplotypes with grain weight, length, and width were observed in three, one, and four environments, respectively (Figure 6C). *Cis*‐acting elements prediction in the promoter of *TaRBL14a‐Hap1* and *TaRBL14a‐Hap2* indicated that the SNP at −779 bp site in the promoter region was one of the components of binding sites to the transcription factor TCP, which has been reported to be associated with spike/grain development (Figure 6A). This suggested that *TaRBL14a‐Hap2* was an elite haplotype for TGW and grain yield in wheat.

### 3.9 *TaRBL14a‐Hap2* was positively selected for wheat breeding in China

**FIGURE 7:**
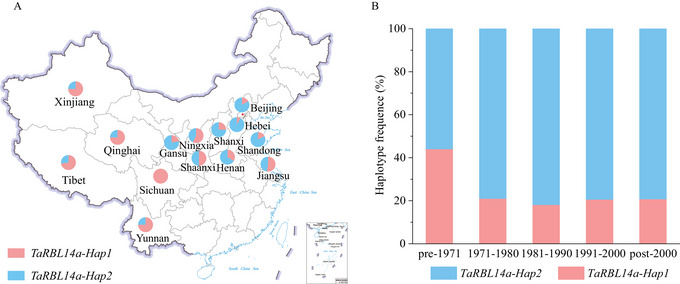
Spatial and temporal distribution of *TaRBL14a* haplotype. (A) Geographic distribution of varieties with *TaRBL14a* haplotypes in China. The map was downloaded in the Standard Map Service System (http://bzdt.ch.mnr.gov.cn/). (B) Frequencies of *TaRBL14a* allelic variation in Chinese wheat breeding programs in different decades.

Artificial selection leads to the gradual accumulation of elite haplotypes during domestication history. To investigate whether the elite haplotype *TaRBL14a‐Hap2* had been positively selected in wheat breeding, we evaluated the geographical distribution of two haplotypes of *TaRBL14a* in China using 325 wheat varieties from 14 provinces in China (Figure 7A; Table S7). The results showed that the accessions with *TaRBL14a‐Hap2* were the dominant accessions in Hebei (91%), Henan (66%), Shandong (83%), and Shanxi (75%), which were the main wheat‐producing areas in China. To further determine whether haplotype *TaRBL14a‐Hap2* was positively selected during wheat breeding in China, we used 321 different wheat varieties to analyze the selection of allele variants of the *TaRBL14a* gene in wheat during historical breeding (Figure 7B; Table S8). The results showed that *TaRBL14a‐Hap2* was positively selected in wheat domestication history. The spatiotemporal distribution results revealed that the elite haplotype *TaRBL14a‐Hap2* had been positively selected in wheat domestication history for improving wheat grain traits in China.

## 4 DISCUSSION

As the intramembranous proteases, RBL proteins are involved in various cellular functions and development processes through the intramembranous proteolysis (Koonin et al., 2003; Urban & Dickey, 2011). The *RBL* genes have been identified in many high plants, such as *Arabidopsis*, rice, maize, and poplar (*Populus* L.) (García‐Lorenzo et al., 2006; Li et al., 2015; Roman & Kaldunski, 1991), but a comprehensive analysis of *RBL* genes has not yet been performed at the genome‐wide level of wheat. In this study, we identified 41 members of the *TaRBL* family and classified them into five subfamilies: RhoA, RhoB, RhoC, RhoD, and RAPL. Most *TaRBL* genes were highly expressed in spike/grain and could play critical roles in grain development.

In plants, there were differences in the copy numbers of *RBL* genes, ranging from 4 in green alga (*Ostreococcus lucimarinus*) to 13 in rice, 17 in *Arabidopsis*, and 41 in wheat (Li et al., 2015). After gene duplication, the members of the *RBL* family lost or changed their genetic information during their evolution. This results in differences in amino acid length and the functional diversity of the RBL protein. For instance, the difference in the catalytic activity between AtRBL2 and AtRBL1 in *Arabidopsis* resulted in AtRBL2 being able to cleave the drosophila rhomboid substrates Spitz and Keren (Kanaoka et al., 2005). The phylogenetic analysis revealed that the variations in plant copy number were primarily attributed to the RhoA subfamily. The fragment duplication gene pairs were predominantly found in the RhoA subfamily, which could account for these differences. The regulatory genes, that is, transcriptional and developmental regulators, and signaling genes were more likely to be retained after duplication events, consistent with the previous report that most RhoA‐type *RBL* genes were primarily involved in the signaling‐related regulatory processes (Lei et al., 2012; Maere et al., 2005; Van de Peer et al., 2009).

Subcellular prediction results showed that TaRBL proteins exhibit a wide distribution in various cellular organelles, including mitochondria, chloroplasts, Golgi apparatus, and ER. Alterations in the subcellular localization of these proteins may consequently cause functional differentiation. For example, AtRBL2 was found to localize on the Golgi apparatus and cleave the Drosophila ligands Spitz and Keren, when expressed in mammalian cells (Kanaoka et al., 2005). AtRBL10 was located in the chloroplasts and was involved in floral development and fertility (Thompson et al., 2012). A recent study reported that the ANAC013‐RBL2 module localized in the ER played a role in the initial reaction phase of *Arabidopsis* hypoxia, *rbl* knockout mutants exhibited impaired low‐oxygen tolerance (Eysholdt‐Derzsó et al., 2023). In this study, we found that both TaRBL14a and TaRBL14b contained the zf‐RanBP domain at their C‐terminus, which were predicted to be localized on the plasma membrane and specifically expressed in spike. The C‐terminus of TaRBL14c, TaRBL14d, and TaRBL14e contained ZnF_RBZ domains, which were predicted to be located on chloroplasts and mainly expressed in leaf, spike, and grain (Nakielny et al., 1999; Schultz et al., 1998; Yaseen & Blobel, 1999). These results explained that different subcellular localization, sequence structure, and expression patterns implied functional differentiation of proteins within the same family.

The analysis of promoter *cis*‐acting elements and tissue localization expression patterns suggested that most of the *TaRBL* family were associated with the development of wheat spike/grain. In rice, inhibiting the expression of *OsDER1* led to decreased GL and width via changing starch and protein body I morphology (Qian et al., 2018). In *Arabidopsis*, the RBL8 affected the morphological changes of pollen outer wall, and RBL10 plays a role in floral development (Kanaoka et al., 2022).

A higher grain yield is an important goal for wheat breeders. Marker‐assisted selection (MAS) plays a crucial role in improving wheat crops through breeding (L. Guo et al., 2022; Khan et al., 2022). The increase in wheat yield largely depends on higher TGW (Ur Rehman et al., 2019). Our results showed that *TaRBL14a‐Hap2* was associated with higher TGW, GL, and GW compared to *TaRBL14a‐Hap1*. This suggested that *TaRBL14a‐Hap2* was an elite haplotype for improving wheat yield. Given that the elite haplotype of *TaRBL14a* was actively selected in the main wheat‐producing areas and breeding history, further selection of the elite haplotype might be helpful for the continuous improvement of wheat grain yield. In this study, the KASP molecular marker developed for *TaRBL14a* provides an important function marker for improving TGW by using the marker‐assisted selection (MAS) strategy in future wheat breeding.

In this study, qRT‐PCR results indicated that *TaRBL* genes could participate in response to drought stress, salt stress, and ABA treatment. It has been reported that the ANAC013‐RBL2 module localized in the ER and played a role in the initial reaction of hypoxia in *Arabidopsis* (Eysholdt‐Derzsó et al., 2023). *RBL* genes have also been shown to be involved in ABA signaling transduction (Li et al., 2015). Transgenic rice with *OsDER1* overexpression or suppression showed hypersensitivity to ER stress with increased levels of ubiquitinated proteins (Qian et al., 2018). These results provided insights for further exploration of the function of the *RBL* genes. Therefore, *TaRBL* genes may serve important roles in response to diverse abiotic stress.

## 5 CONCLUSIONS

A total of 41 *TaRBL* genes were identified and divided into five subfamilies with potentially similar functions. Fragment duplication emerged as the primary driver of *TaRBL* family genes expansion. The results of qRT‐PCR showed that *TaRBL* genes were highly expressed in young spikes and differentially expressed under PEG, NaCl, and ABA treatments, suggesting that they were involved in spike/grain development and abiotic stress response. *TaRBL14a‐Hap2* was an elite haplotype for TGW and grain yield in wheat. These findings will provide new insights into further exploring the biological function of *RBL* genes and potentially applying the elite haplotype in wheat breeding program.

## AUTHOR CONTRIBUTIONS

**Yanyan Zhang**: Data curation; formal analysis; validation; visualization; writing—original draft. **Xiaoya Huang**: Formal analysis; investigation. **Long Zhang**: Formal analysis; investigation. **Weidong Gao**: Investigation; visualization. **Jingfu Ma**: Data curation; software. **Tao Chen**: Conceptualization; funding acquisition; resources; writing—review and editing. **Delong Yang**: Conceptualization; funding acquisition; resources; writing—review and editing.

## CONFLICT OF INTEREST STATEMENT

The authors declare no conflicts of interest.

## DATE AVAILABILITY STATEMENT

The datasets used and/or analyzed during the current study are available from the corresponding author on reasonable request. Some of the data are shown in Supporting Information.

## Supporting information




**Table S1**. Identified motifs of RBLs in wheat.
**Figure S1**. Multiple sequence alignments of wheat RBL proteins, with the predicted approximate transmembrane helices marked with black lines, with the red line indicating the motif and the red dotted box indicating the important motifs.
**Figure S2**. Chromosomal distributions of *TaRBLs*. The scale for the chromosomal length is millions of bases (Mb).
**Table S2**. Ka/Ks ratios for duplicated *TaRBL* gene pairs.
**Figure S3**. Functional classification of predicted *cis*‐elements in *TaRBLs* promoter regions.
**Figure S4**. The *cis*‐acting elements related to development identified in the promoter region of each *TaRBLs*.
**Figure S5**. The *cis*‐acting elements related to stress responsive identified in the promoter region of each *TaRBLs*.
**Figure S6**. The *cis*‐acting elements related to phytohormone identified in the promoter region of each *TaRBLs*.
**Figure S7**. Heat map showing the expression profile of *TaRBLs* in different organs of wheat and their three development stages. Heat map was created by using log10‐transformed FPKM expression values. Color green or red demonstrates lower and higher expression levels, respectively. Z10: One‐leaf stage; Z13: Three‐leaf stage; Z23: Early tiller stage; Z30: Booting stage; Z32: Early jointing stage; Z39: Late jointing stage; Z65: Mid‐flowering stage; Z71: 2 d after flowering; Z75: 10 d after flowering; Z85: 30d after flowering.
**Figure S8**. Detection of 10 wheat accessions containing two haplotypes. A. Sequence amplification based on ‐779 bp (C/G). 1–5 represent the varieties with the first haplotype, including Shi 4185, Liangxing 99, Lumai 14, Jimai 26, and Lunxuan 987. 6–10 represent the varieties with the second haplotype, including Hengguan 35, Mazhamai, Xinong 6028, Zhongyou 9507, and Jinmai 47. Allelic variation C (B) and G (C) of *TaRBL14a* at different materials ‐779 bp.
**Figure S9**. Association of *TaRBL14a‐Hap1* and *TaRBL14a‐Hap2* with TGW, GL, GW and GT of 111 wheat accessions at three environments allelic. * *p* < 0.05; ** *p* < 0.01.
**Table S3**. Primer sequences used in this study.
**Table S4**. TaRBL14a‐KASP data based on ‐779 bp in wheat accessions collected from the different areas.
**Table S5**. Wheat accessions information from published study (left) and collected from different areas (right) for association analysis.
**Table S6** Grain phenotypic traits for association analysis.
**Table S7**. The information of the wheat diversity panel and their genotypes distribution of *TaRBL14a*.
**Table S8**. The information of the wheat diversity panel and their genotypes of *TaRBL14a* gene in the different decades.

Supporting Information
