# Supplementary material for: Genome‐wide identification, gene expression and haplotype analysis of the rhomboid‐like gene family in wheat (Triticum aestivum L.)
Source: Plant Genome. 2024 Feb 13;17(2):e20435. doi: 10.1002/tpg2.20435 (PMC12807219; doi:10.1002/tpg2.20435)
Supplement: Supplementary file 1 [file TPG2-17-e20435-s002.docx]

TMH 1

motif 5

TMH 1

motif 19

motif 18

motif 5

motif 19

motif 2

motif 20

motif 12

motif 16

motif 12

motif 8

motif 6

motif 13

motif 14

motif 7

motif 10

motif 4

TMH 4

TMH 5

TMH 6

TMH 3

TMH 2

motif 1

motif 10

motif 9

motif 20

motif 2

TMH 7

motif 11

motif 15

motif 3

motif 13

motif 17

motif 11

motif 6

motif 15

motif 3

Supplementary Figure 1

Supplementary Figure 2

Supplementary Figure 3

Supplementary Figure 4

Supplementary Figure 5

Supplementary Figure 6

Supplementary Figure 7

Supplementary Figure 8

Supplementary Figure 9
